# Cell cycle progression is regulated by intertwined redox oscillators

**DOI:** 10.1186/s12976-015-0005-2

**Published:** 2015-05-29

**Authors:** Jorgelindo da Veiga Moreira, Sabine Peres, Jean-Marc Steyaert, Erwan Bigan, Loïc Paulevé, Marcel Levy Nogueira, Laurent Schwartz

**Affiliations:** Ecole Polytechnique, LIX–UMR 7161, Palaiseau, France; LRI, Paris-Sud University, CNRS UMR8623 and INRIA Saclay, Paris, France; Paris Institute of Translationnal Neurosciences (IHU-A-ICM), Pitié Salpêtrière Hospital, Paris, France

**Keywords:** Cell cycle, CCM, REDOX, Intracellular pH, ATP/ADP, NAD(P)^+^/NAD(P)H, HATs, HDACs

## Abstract

The different phases of the eukaryotic cell cycle are exceptionally well-preserved phenomena. DNA decompaction, RNA and protein synthesis (in late G_1_ phase) followed by DNA replication (in S phase) and lipid synthesis (in G_2_ phase) occur after resting cells (in G_0_) are committed to proliferate. The G_1_ phase of the cell cycle is characterized by an increase in the glycolytic metabolism, sustained by high NAD^+^/NADH ratio. A transient cytosolic acidification occurs, probably due to lactic acid synthesis or ATP hydrolysis, followed by cytosolic alkalinization. A hyperpolarized transmembrane potential is also observed, as result of sodium/potassium pump (NaK-ATPase) activity. During progression of the cell cycle, the Pentose Phosphate Pathway (PPP) is activated by increased NADP^+^/NADPH ratio, converting glucose 6-phosphate to nucleotide precursors. Then, nucleic acid synthesis and DNA replication occur in S phase. Along with S phase, unpublished results show a cytosolic acidification, probably the result of glutaminolysis occurring during this phase. In G_2_ phase there is a decrease in NADPH concentration (used for membrane lipid synthesis) and a cytoplasmic alkalinization occurs. Mitochondria hyperfusion matches the cytosolic acidification at late G_1_/S transition and then triggers ATP synthesis by oxidative phosphorylation. We hypothesize here that the cytosolic pH may coordinate mitochondrial activity and thus the different redox cycles, which in turn control the cell metabolism.

## Background

For several years now, a number of studies have been conducted, in the field of the bioenergetic origin of life [1]. Bioenergetics consists in converting nutrients such as carbohydrates, lipids, and proteins, into intermediate metabolites as well as energy for cell survival and finally into *de novo* building blocks such as nucleic acids, proteins and lipids for cell proliferation. More broadly, cell metabolism is the sum of all the chemical reactions and dynamic exchanges between a cell and its microenvironment. Eukaryotic cells, at least, exhibit two opposite metabolisms: anabolic reactions, which consist in biomass synthesis and catabolic reactions, leading to the breakdown of macromolecules for energetic use. These two aspects of cell metabolism are managed by biochemical and biophysical oscillators, including reductive and oxidative (redox) couples, the most important ones being Nicotinamide Adenine Dinucleotide (NAD^+^/NADH) and Nicotinamide Adenine Dinucleotide Phosphate (NADP^+^/NADPH), the universal energy carrier, Adenine Triphosphate (ATP/ADP), the transmembrane potential (Vm) and, last but not least, the intracellular pH (pHi) of the cell. The dynamics of these internal biological rhythms are shown to exhibit oscillatory phenotypes in dividing cells [2].

**Fig. 1:**
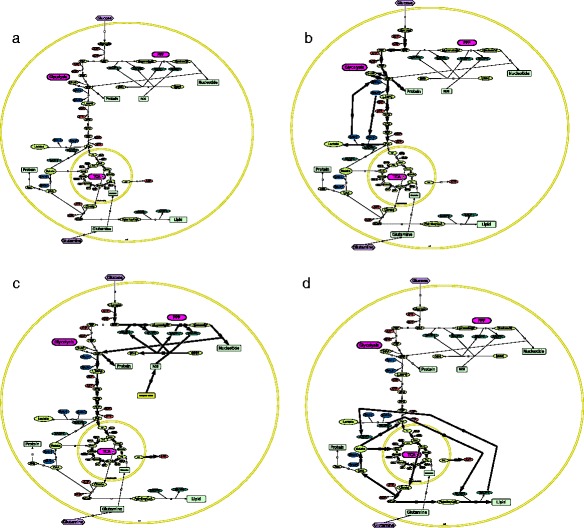
The central carbon metabolism (CCM). **a** The different phases of the eukaryotic cell cycle could be explain by reductive-oxidative (redox) transitions in the CCM. **b** In G1, high ATP demand for protein synthesis is managed by anaerobic glycolysis leading to lactate synthesis. This permits NAD+ regeneration by lactate dehydrogenase and then sustains high NAD+/NADH ratio. **c** In S phase, the CCM is shifted to pentose phosphate pathway (PPP) for nucleotide synthesis. **d** In G2 the tricarboxylic acid (TCA) pathway and the electron transport chain are fully active and allow mitochondrial ATP synthesis and lipid synthesis from citrate

The intriguing metabolic feature of proliferating cells compared with quiescent ones highlights the well-conserved sequential events characterizing the eukaryotic cell cycle. From the point of view of the central carbon metabolism (CCM), (Fig. 1), the quiescent cells (in G_0_) have a basal oxidative metabolism, whereas, in proliferating cells, the carbon flux is rewired to biomass synthesis and cell growth [3]. The latter is enhanced by a high glycolytic rate consuming NAD^+^ and ADP species for cytoplasmic glucose conversion into pyruvate, generating NADH and ATP molecules. NADH is oxidized back to NAD^+^ through pyruvate conversion into lactate, termed as the Warburg effect after the German Nobel laureate Otto Warburg, and ATP is used as an energy supplier for RNA and protein synthesis in G_1_ of the cell cycle. Glycolysis is then shunted to the pentose phosphate pathway (PPP), generating nucleic acid precursors for DNA replication in the S phase and NADPH reductive species used later on in the cell cycle progression for membrane lipid synthesis in G_2_. The G_2_ phase is also characterized by full mitochondrial activity, where the citric acid cycle takes place, enabling glucose oxidation and ATP synthesis.

Moreover, current hypothesis support the proton gradient-dependent ATP hydrolysis and synthesis into the cytosol and mitochondria, respectively, as critical events in both transmembrane potential and intracellular pH oscillation during cell cycle. In this study we aim at juxtaposing interesting results confirming the hypothesis of the pivotal role of pHi on mitochondrial activity and the resulting redox oscillations timing the progression of the cell cycle. For that, we first report the redox phenomena involved in central carbon metabolism and how it regulates the metabolic transitions during the cell cycle progression. Secondly, based on literature reports, we highlight intracellular pH role in cell metabolism and its potential involvement in “clocking” transitions during the cell cycle.

### Cellular redox transitions in CCM during cell cycle progression

#### 1. The metabolic status of quiescent cells in G_0_

**Fig. 2:**
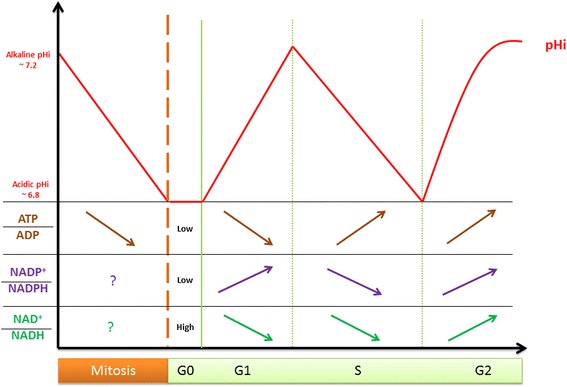
The logic of metabolic cell cycle. Cytosolic NAD+/NADH, NADP+/NADPH, ATP/ADP ratios and intracellular pH (pHi) are reported to oscillate through eukaryotic cell cycle. Mitosis is often described as a catastrophic event where microtubule depolymerization and ATP-dependent pumps « burn » the ATP stock by ATP hydrolysis. This decreases the ATP/ADP ratio. In parallel, the intracellular pH is reported to decrease and reaches its stationary phase in quiescent cells (G0). To our knowledge, there are no data on NAD+/NADH and NADP+/NADPH redox ratios during mitosis. In early G1 phase the increased glycolytic pathway matches a transient acidic pHi before cytosol alkalinization. This is often assumed to be linked to lactate synthesis. Lactate dehydrogenase enzyme catalyses pyruvate conversion to lactic acid by using NADH as coenzyme. NAD+/NADH ratio is high in G1 and decreases until reaching its minimal value in S phase. NADPH consuming pathway such as ROS conversion to reduced species is known to trigger cell cycle entry and enhances PPP by increasing the NADP+/NADPH redox ratio in S phase. This is a necessary step for nucleic nucleotide synthesis. During this phase cytosolic acidification, probably due to glutaminolysis, meets hyperfused mitochondria where ATP synthesis takes place. NAD + −dependent histone synthesis is thought to decrease the NAD+/NADH ratio. In G2, both free ATP concentration and pHi reach their maximal value, high and alkaline, respectively. NADPH consumption during fatty acids synthesis may increase NADP+/NADPH ratio in G2. NAD+/NADH ratio is reported to increase in G2. This could be explain by increased shuttling pathways such as malate/citrate one, permiting NAD+ synthesis from oxaloacetate to malate conversion

Quiescent cells have a basal oxidative metabolism [3]. They use nutrients such as glucose, protein and fatty acids as main energy supplier to support primary reactions such as amino acids and nucleotide synthesis (see Fig. 1-a) [4]. For that, the glycolytic pathway converts glucose to pyruvate and produces the universal energy transporter in living systems, adenosine triphosphate (ATP). Pyruvate can either be converted into lactate, in the cytosol, or join the tricarboxylic acid (TCA) cycle, taking place in mitochondria. There, ATP is synthesized by oxidative phosphorylation. On the other hand, macromolecules such as proteins and fatty acids are also degraded and join mitochondria for full conversion to ATP, carbon dioxide (CO_2_) and water [3]. The electrochemical energies released during these catabolic reactions are captured by electron carrier species such as nicotinamide adenine nucleotide (NAD^+^), converting it to its respective reduced partner (NADH,H^+^). NADH,H^+^ is the electron donor of the mitochondrial electron transport chain (ETC.) during oxidative phosphorylation. It is not clear if NADH is able to cross the mitochondrial membrane. However, it is reported that specific shuttles such as the malate/citrate shuttle allows mitochondrial NADH regeneration through the TCA cycle [5] (see Fig. 1-a). This basal metabolism allows homeostatic control of high NAD^+^/NADH and low NADP^+^/NADPH redox ratios [6] in resting cells. On the opposite, in proliferating cells, the basal catabolic metabolism is shifted to anabolism and exhibit oscillatory conversion of these redox species for biomass synthesis and cell growth [3] (see Fig. 2).

#### 2. Redox oscillation in dividing cells

##### 2.1. Cell cycle entry into G1 is regulated by the Warburg effect

Glycolysis is one of the fundamental pathways of living organisms. It allows the conversion of one molecule of glucose into two pyruvates. During this process, universal energy transporters, such as ATP and NADH, are produced from the oxidative conversion of ADP and NAD^+^, respectively. This pathway is characterized by two phases: the so-called “investment” phase, since it consumes two ATP molecules, and the “payoff” phase, which produces four ATP. The first phase consists in catabolizing one molecule of glucose into two carbon phosphate derivatives, glyceraldehyde 3-phosphate (G3P). The second one gives two pyruvates that will later play a pivotal role in mitochondria-dependent energy production. In one study, Diaz-Moralli and colleagues (2012) assume that the glycolytic pathway mainly occurs during the first growth phase of the eukaryotic cell cycle (G_1_) [7]. Indeed, in G_1_ cells grow and synthesize messenger RNA (mRNA) for protein synthesis. Moreover, these studies [8–10] reported the aerobic glycolysis to follow biochemical principles based on “thermodynamic favorability, availability of enzymatic mechanisms and the physicochemical properties of pathway intermediates”, meeting the cells’ energy demands for cell proliferation.

High glycolytic flux in central carbon metabolism (CCM) occurs when quiescent cells are committed to proliferation [3]. High ATP demand for protein synthesis meets high NAD^+^/NADH redox ratio, allowing glycolysis to persist through the fermentative pyruvate to lactate conversion by lactate dehydrogenase enzyme (Fig. 1-b). This consists in a metabolic switch from oxidative phosphorylation, in quiescent cells, to glycolytic phosphorylation, in non-transformed proliferating cells, entering cell cycle [11, 12]. Calderon-Montano and colleagues [11] highlighted the intracellular pH (pHi) role in regulating glycolytic genes such as phosphofructokinase-1 (PFK-1). Increased pHi such as observed in cancer cells increases biomass (DNA and protein) synthesis. This anabolic metabolism observed in rapidly-proliferating cells results in redox oscillations of cytoplasmic free ATP concentration, as well as the NAD^+^/NADH ratio [13, 14] (Fig. 2). It sustains the aerobic glycolysis observed in G1, just as described by Otto Heinrich Warburg in its seminal work on the glycolytic mode of cancer metabolism [15]. Interestingly, recent studies provide explanations of the Warburg effect, from quantitative models of the metabolic shift in cancer cells, which has the same metabolic signature as normal-proliferating cells in G1 phase [16–18]. These are based on analytic rules deciphering the dualistic aspect of proliferating cells metabolism where the abundant resource triggering cell cycle entry favors the glycolytic phenotype, referred as Warburg effect in cancerous tissue. In short, the Warburg effect drives the volumetric growth in G1 by “metabolosomic” biomass synthesis whereas mitochondria activity triggers the surfacic growth.

It is noteworthy that this biphasic growth taking place in proliferating cells is under the control of growth stimuli and redox species oscillation [19, 20]. In mammalian cells, growth factors and their respective receptors are reported to generate ROS and trigger cell cycle entry [7, 21, 22]. Therefore, moderate ROS formation in late G_1_ is essential for gene transcription and protein synthesis, by modulating DNA accessibility [7]. In fact, an extensive number of studies support that histone acetylation is a pivotal epigenetic program controlling eukaryotic gene transcription [23–25]. In short, transcriptionally active genes are shown to meet hyperacetylated histones, while the “hypoacetylated histone is associated with transcriptionally repressed genes” [25, 26]. Firstly, it is shown that the cytoplasmic NAD^+^/NADH redox ratio optimizes the glycolytic flux (Warburg effect) to ATP and amino acid synthesis. Secondly ATP is used as a supplier of energy for gene transcription and protein synthesis, using these amino acids as building blocks.

##### 2.2. NADP^+^/NADPH ratio regulates the pentose phosphate pathway

In normal proliferating cells, one of the first pathways switched on after aerobic glycolysis, is the pentose phosphate pathway (PPP) (Fig. 1-c). This is the main circuit for DNA precursor synthesis and nicotinamide adenine dinucleotide phosphate (NADPH) regeneration, a coenzyme used by the cell for lipid synthesis, in G_2_, and plays the role of reactive oxygen species (ROS) scavenger in G_1_/S transition phase [7, 27–29]. The PPP is characterized by two branches: the oxidative branch, where NADP^+^ is reduced to NADPH by the shunt of glycolysis from glucose 6-phosphate (G6P) conversion into ribulose 5-phosphate (R5P) [30], and the non-oxidative branch rewiring the PPP to aerobic glycolysis up to fructose 6-phosphate (F6P) and glyceraldehyde 3-phosphate (G3P).

Interestingly, the PPP has been shown to be regulated by the NADP^+^/NADPH ratio [27, 31, 32]. Therefore, glucose 6-phosphate dehydrogenase (G6PDH) enzyme, catalyzing the conversion of G6P into 6-phosphogluconolactone, has been shown to have an allosteric activity, modulated by the cytoplasmic NADP^+^/NADPH ratio [31]. This is in agreement with these studies [27, 32], which assume that the G6PDH catalytic activity is accelerated by an increased NADP^+^/NADPH ratio, a result of cellular NADPH consumption by reactive oxygen species (ROS) in S phase.

In this study [29] on changes in the activity of the PPP, the authors showed that NADPH-consuming pathways enhance the pentose phosphate cycle (Fig. 2-c). At least two significant pathways have been identified as major NADPH consuming systems: the glutathione redox cycle (GSH/GSSH) and the lipid synthesis pathway [6, 33, 34]. The reduced glutathione (GSH) is a protective and antioxidant agent that reduce reactive oxygen species (ROS), used in signaling cascades to trigger cell cycle entry (see above) [7], and generates the oxidative species (GSSH). NADPH reduces GSSH and, as a result, they both are converted back to their respective oxidative “partner”, GSH and NADP^+^. In doing so, the glutathione redox cycle enhances the primary pathway using NADP^+^ as a coenzyme: the PPP oxidative branch. The second NADPH-consuming pathway is lipid synthesis, which builds fatty acid blocks from acetyl-CoA.

#### 3. Mitochondria and their pivotal role in cell anabolic demand in G_2_

Mitochondrial activity is a key in cell metabolism decision-making and cell cycle progression. As it has been extensively reviewed in this study [35], mitochondria are organelles that have been the subject of many controversies. They were first considered as just a “powerhouse” of the eukaryotic cell, before the pioneering studies deciphering their key role in processes such as development, survival, division, and cell death. Regarding cell division, investigations support and highlight the idea of intertwined relationships between machineries governing mitochondrial dynamics and cell cycle metabolism [36, 37]. It has then been demonstrated that energy transitions occurring in the cell cycle are intrinsically linked to mitochondria sensing parameters, such as the intracellular pH (pHi) [38] and ATP/ADP ratio [39–41].

Recent studies highlight the mitochondrial morphogenesis at the G_1_/S transition of the cell cycle. It is also shown that at the G_1_/S checkpoint, mitochondria form a single giant factory for ATP synthesis [5, 42]. “This energetic boost” is thought to be necessary to increase cyclin E expression in order for the cell to pass the G_1_/S checkpoint [41]. As stated above, this event could also be interpreted as a necessary step in cell energy supply for protein, nucleic acid, and membrane lipid synthesis (Fig. 1-d). Interestingly enough, these bodies of works support the idea of cell cycle progression meeting the tricarboxylic acid (TCA) cycle and oxidative phosphorylation in S/G_2_ phase transition [7]. The TCA or Krebs cycle, which takes place in mitochondria, in conjunction with oxidative phosphorylation, does indeed allow for carbohydrate oxidation to CO_2_, H_2_O and TCA intermediate species. This is also the most efficient route for ATP and lipid precursors’ synthesis in the mitochondrial matrix.

The pyruvate accumulated in the cytosol from glycolysis passes through the recently identified specific mitochondrial pyruvate carrier (MPC) and is converted into acetyl-CoA [43–45]. This reaction is catalyzed by pyruvate dehydrogenase with NAD^+^ as coenzyme. The first step in the TCA cycle is acetyl-CoA conversion into citrate through dehydration. According to the metabolic state of the cell, citrate can shunt to lipid synthesis or continue the Krebs cycle (Fig. 1-d). For that, key enzymes are regulated by negative feedback loops [46]. This includes citrate synthase, isocitrate dehydrogenase and α-ketoglutarate dehydrogenase. These enzymes are down-regulated by NADH and ATP. Also, this half part of the TCA cycle is the main route for glutaminolysis. This consists of a series of biochemical reactions by which the glutamine amino acid is lysed into glutamate and then α-ketoglutarate. From there, there are two possibilities: the oxidative route enables the full Krebs cycle and conversion of α-ketoglutarate into succinyl-CoA [47]. The reductive route is the α-ketoglutarate conversion to isocitrate and then citrate, the precursor of lipid synthesis. It is interesting to note the reported negative regulation of citrate synthase by succinyl-CoA [46]. Also noteworthy here, is that reductive versus oxidative metabolism mutually exclude each other [48]. At the same time, ATP synthesis through the oxidative phosphorylation chain is required for membrane lipid synthesis in the second growth phase (G_2_) (see Fig. 1-d) [7].

**Fig. 3:**
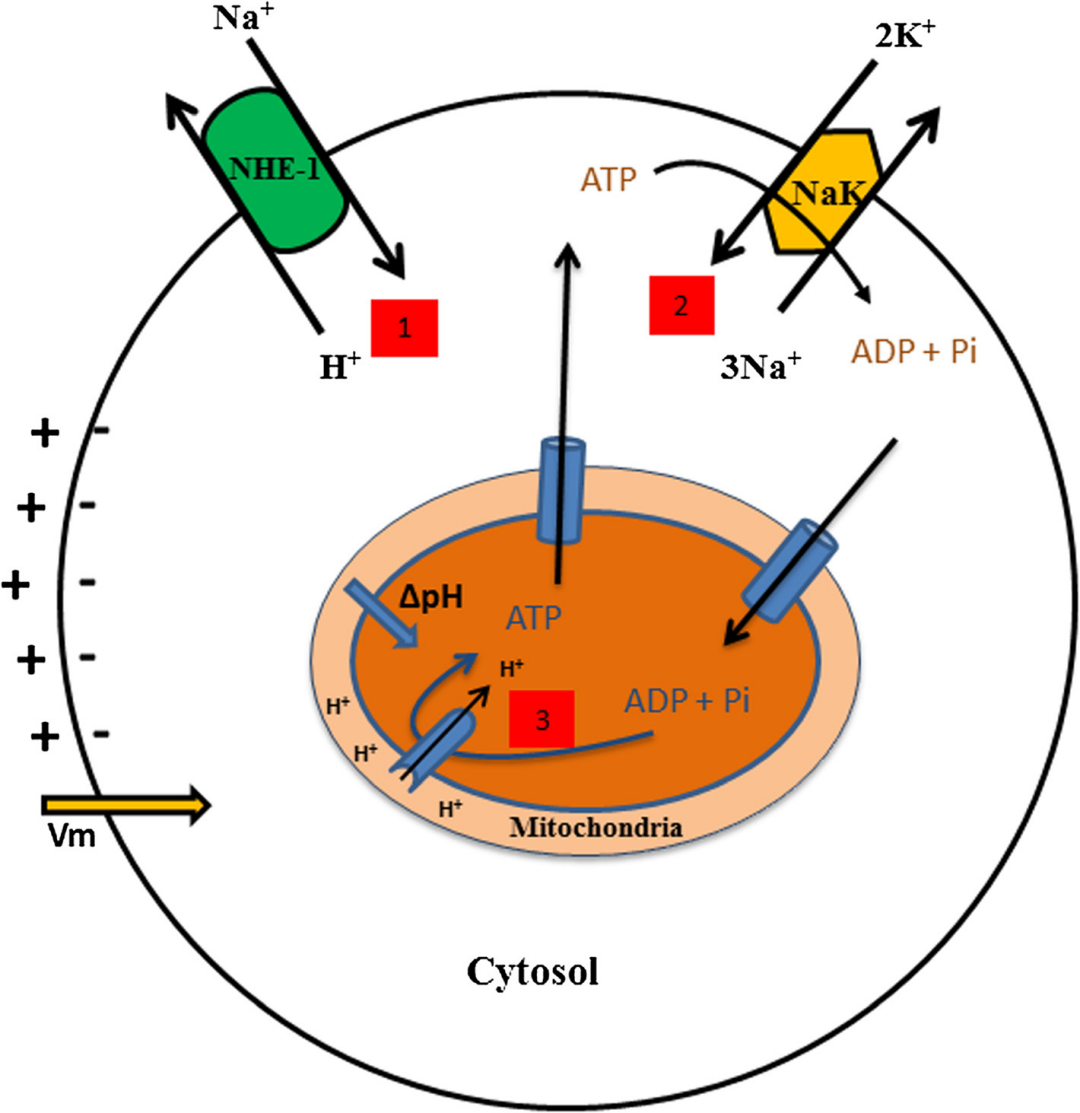
Intertwined relationship between ATP/ADP ratio, the intracellular (pHi) and the transmembrane potential (Vm). Sodium-proton exchanger (NHE-1) which extrudes a proton (H+) against a sodium (Na+) is known to fight further cytosolic acidification. The sodium-potassium electrogenic exchanger (NaK-ATPase), one of the main consumer of ATP in eukaryotic cells, extrudes 3 sodium (Na+) from the cytosol against 2 inward potassium (K+) flux and creates an hyperpolyrisation of the cell membrane. Finally, Increased ΔpHi from mitochondria matrix and intermembrane space is reported to increase ATP synthesis

Oxidative phosphorylation mainly occurs in the internal membrane of the mitochondria in eukaryotic cells. It uses TCA cycle precursors and co-enzymes, such as NADH and FADH_2_, as electron donors for respiration. Through a cascade of reactions, the ETC. complexes trigger the pumping of protons out of the matrix and enable a pH gradient (ΔpH) required for ATP synthesis (Fig. 3) [49]. In this redox chain, molecular oxygen (O_2_) is used as the ultimate electron acceptor and ATP is generated as protons moves down its concentration gradient through a well evolutionary-conserved enzyme called ATP synthase, in the inner membrane of mitochondria (reviewed here [49]. This chemiosmotic theory, developed by P. Mitchell [50], explains how NADH and FADH_2_ oxidation are coupled to ADP phosphorylation into ATP. This is a coupling between oxidation and phosphorylation by a proton gradient across the inner mitochondrial membrane. The oxidative energy from NADH is converted into osmotic energy by proton gradient formation across the membrane, where the intermembrane space is more acidic and the matrix is alkaline. The ΔpH, which is the pH difference between the matrix and the intermembrane space, is generated by enzymatic complexes of the ETC. Five complexes have been identified. Only complexes I, III, IV are proton extruders. Four protons are extruded by complexes I and IV and two protons for complex II. The last complex is the ATP synthase catalyzing ADP phosphorylation into ATP against three protons diffusing back to the mitochondrial matrix.

As stated above, the mitochondrial respiratory chain is mainly fueled by the NAD^+^/NADH redox couple. In order for the proliferative cell to maintain high ATP synthesis for cytoskeletal dynamics and membrane lipid synthesis, a symbiotic structure is formed between cytosol and the mitochondria. These are the metabolic shuttles, where one of the most important ones being the malate/citrate shuttle (Fig. 1-d). In fact, while mitochondrial ATP synthesis is high in G_2_, citrate is shuttled out from the Krebs cycle [7, 13]. In the cytosol it is converted into acetyl-CoA and oxaloacetate [3, 51]. As mentioned in the sections above, acetyl-CoA is used as a fatty acid precursor for membrane synthesis and cell growth in G_2_. This well-described pathway consumes NADPH, generated from the malic enzyme and the pentose phosphate pathway [6]. On the other hand, oxaloacetate is converted into malate by consuming NADH and produces NAD^+^. The reverse reaction occurs in mitochondria, where malate, shuttled-in from cytosol, is converted back to oxaloacetate during the Krebs cycle and generates NADH, the first electron donor of ETC. This forms a full cycle in G_2_ (Fig. 1-d), where NAD^+^/NADH and NADP^+^/NADPH redox ratios both increase in order to fulfill lipogenesis.

The last but not least cycling parameter of the metabolic cell cycle is intracellular pH, which has been reported to match mitochondrial activity and described as a potential “internal clock” for cell mitosis [52].

### Intracellular pH and ATP/ADP ratio time the metabolic cell cycle

#### 1. ATP concentration oscillations in proliferating cells

In their study, Martin and Müller [53] hypothesized that the eukaryotic cells’ common ancestor arose from a symbiotic process between an anaerobic, autotrophic and “strictly hydrogen-dependent *archaebacterium*” and a respiring *eubacterium,* releasing hydrogen “as a waste product of anaerobic heterotrophic metabolism”. This symbiosis between the hydrogen-dependent host and the symbiont, which produce hydrogen, is an attractive hypothesis supporting P. Mitchell’s chemiosmotic theory of oxidative phosphorylation-dependent ATP synthesis in mitochondria [49]. In this model, it is assumed that the proton gradient across the internal membrane drives the electron transport through the ETC. As reported above, this is performed by transmembrane complexes, which pump protons from the matrix to the intermembrane space. Then this gradient triggers ATP synthase activity and ATP synthesis.

Further, studies explain the energy of the cell from [ATP]/[ADP] ratio point of view. Understanding the bioenergetics of a normal dividing cell at the scale of the entire cell cycle may bring some interesting answers to the cancer cell phenotype [7]. Interestingly, independent studies reported the oscillation of the intracellular ATP concentration through the cell cycle [13] and its extensive use in ionic pump activity [54]. Marcussen and colleagues (1992) reported on ATP concentration oscillation along the progression of the cell cycle [13]. They found that the ATP concentration is minimal at the G_1_/S phase transition and then progressively reaches its maximum at G_2_/Mitosis (Fig. 2). This correlates with the extensive studies by Boonstra and colleagues who reported ATP hydrolysis-dependent Na^+^-K^+^ATPase pump activity during the G_1_/S transition cell cycle [54, 55]. This pump is known to be responsible for the electric potential of large populations of cells by extruding three Na^+^ and against the two K^+^ influx. This is confirmed here, where Veech and colleagues [55] showed that intracellular ATP hydrolysis is tightly linked to Na^+^-K^+^ATPase pump activity, responsible for the inherent oscillation of the electrical potential of the cell [56] (Fig. 3). Consequently, ATP/ADP ratio oscillation modulated by ATP synthesis in mitochondria and hydrolysis in the entire cytoplasm, throughout the cell cycle, is also linked to another intracellular oscillator, i.e., intracellular pH (pHi).

#### 2. Intracellular pH oscillates in phase with ATP/ADP ratio

Increasing evidences indicate that the intracellular pH (pHi) homeostasis is correlated with cell metabolism and proliferation [57–60]. Aerts and colleagues (1985) experimentally showed an autonomous pHi cycle within a *Dictyostelium* cell. They managed to demonstrate that modulating pHi controls protein synthesis and DNA replication: optimal protein and DNA synthesis being correlated with an alkaline pHi of 7.4. This is confirmed by other studies showing the pHi-dependent enzyme activity [61–63]. In their studies, Busa et al. [62] showed that pHi oscillations are master regulators in the decision-making of *brine shrimp* embryonic cells to enter dormancy or to continue development: the acidic pHi is linked to dormancy, whereas the alkaline pHi is characteristic of development. In 1983, Christen et al. highlighted the intertwined relationship between intracellular pH, ATP hydrolysis, and mitochondrial respiration [38]. At acidic pH, the cytoplasmic activity of dynein ATPase, which is involved in microtubule dynamics, is inhibited and the internal ATP concentration is high, translating optimal mitochondrial respiration. On the other hand, when pHi is alkaline, the cytoplasmic ATP concentration falls, probably due to impaired mitochondrial respiration and increased dynein ATPase activity [64]. The authors concluded on the cytoplasmic pH control of ATP hydrolysis, inhibited in acidic pHi and enhanced in alkaline pHi, and mitochondrial respiration increased triggered in acidic pHi and inhibited along with an increased alkaline pHi.

#### 3. Intracellular pH times cell cycle entry and cell growth

**Fig. 4:**
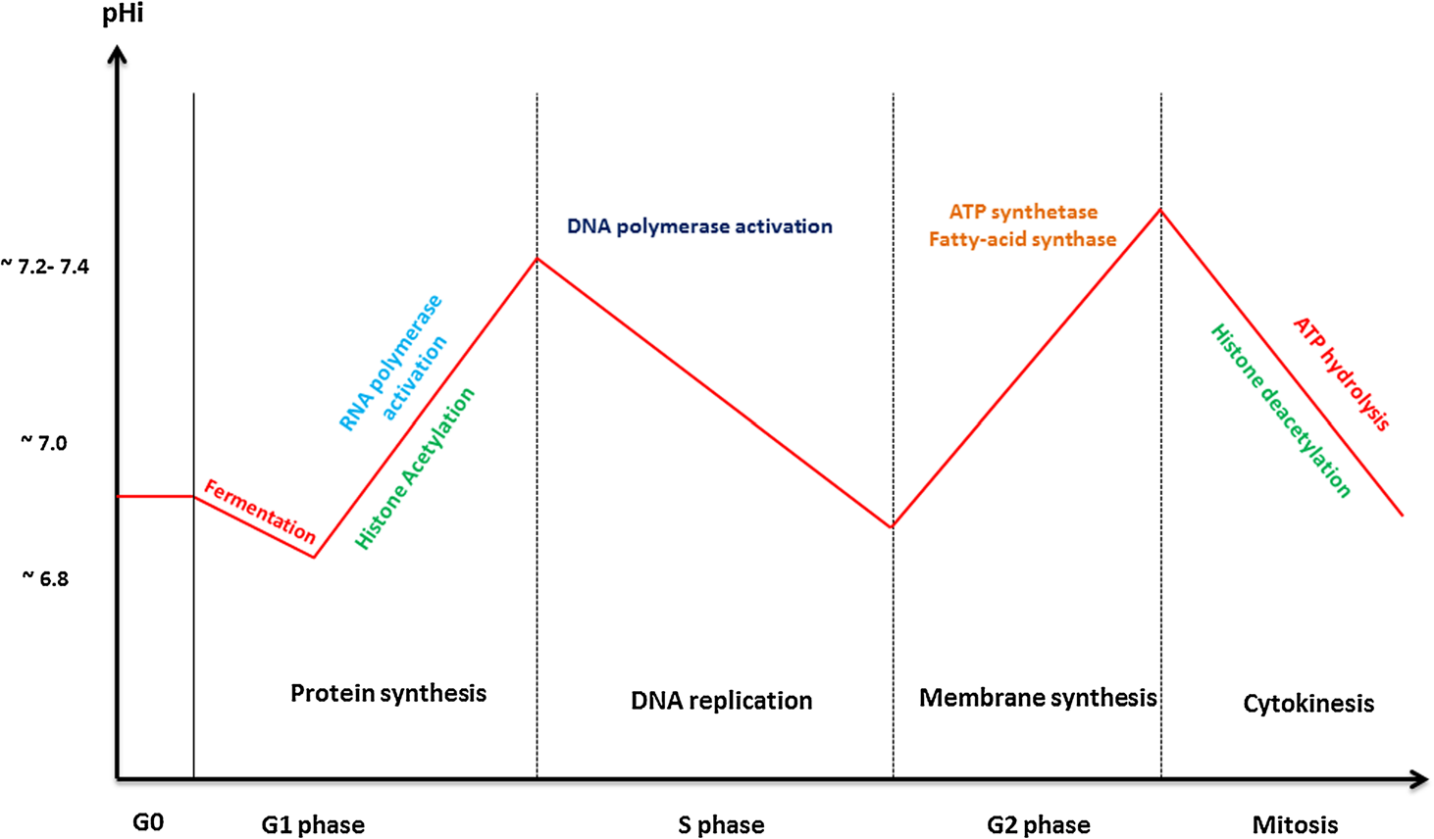
Intracellular pH variation and metabolic activity through the cell cycle. Resting cells have been shown to have a basal oxidative metabolism and a pHi around 7. Cells committed to enter G1 exhibit a transient acidic pH followed by cytosolic alkalinization. Increased pHi has been correlated with histone acetylation allowing for DNA accessibility to RNA polymerase and protein synthesis. A pHi threshold of 7.2 has been reported for DNA synthesis in S phase. This is followed by pHi decrease in late S phase and meet optimal ATP synthesis in mitochondria. Follows lipid synthesis in G2. It has been suggested that a pHi drop during mitosis is linked to microtubule disassembly by ATP hydrolysis. This results in histone deacetylation, DNA compaction and chromosome segregation

The intracellular pH change can be explained by several phenomena [65]. The sodium/hydrogen exchanger, Na^+^-H^+^-1 (NHE-1), plays a significant role, especially in pHi alkalinization [66] (Fig. 3). Moolenaar and colleagues (1981) set up a series of experiments showing the role of NHE-1 in regulating the pHi. Addition of sodium to a neuroblastoma cell culture medium is followed by Na^+^ uptake and H^+^ extrusion. In 2000, Reshkin et al. did transfect normal cells by Human Papillomavirus (HPV), and observed that over-expression of NHE-1 is accompanied by cytoplasmic alkalinity which is first event of carcinogenesis. Recent studies confirm the crucial role of NHE-1 in preventing further cytoplasmic acidification [67–71]. More specifically, NHE-1 activity has been shown to be a key regulator of eukaryotic cell cycle entry and cell growth [69, 72, 73]. Moreover, Pouysségur and colleagues (1985) showed that under growth factor stimulation, the NHE-1 antiporter elevates the cytoplasmic pH of quiescent fibroblasts above a threshold of 7.2, a necessary step for cell cycle entry and DNA synthesis in S phase [63]. Interestingly, some other studies showed a link between the metabolic state of the proliferating cells and histone acetylation [73, 74] (Fig. 4).

Indeed, Histone acetylation plays a pivotal role in regulating gene accessibility to RNA polymerase, for gene transcription [75]. Specific families of enzymes, called Histone Acetyltransferases (HATs) and Histone Deacetylases (HDACs), are responsible for histone acetylation and histone deacetylation, respectively. In this study [73], the authors reported the pivotal role of the NAD^+^/NADH ratio on sirtuin (HDACs) activity. The dynamics of histone acetylation has also been shown to be closely linked to pHi [76, 77]. In this study [76], McBrian and colleagues, showed that histone acetylation has the power of regulating the pHi. The acidic pHi is indeed followed by global histone deacetylation and, thus global histone compaction. This is typically the phenotype of a dormant cell such as the one described above. Conversely, the pHi increase towards alkalinization is reported to favor global acetylation of histone, similarly to when “resting cells are induced to proliferate”. Altogether, these results suggest the intertwined relationship between the metabolic cell cycle balancing the NAD^+^/NADH and ATP/ADP ratios through intracellular pH oscillations.

## Conclusion

Proliferating cells must double their biomass (proteins, lipids, and nucleic acids) through the cell cycle in order to generate two daughter cells. For that they use the central carbon metabolism (CCM), universally shared among living systems. The CCM is governed by pivotal metabolic pathways such as glycolysis, the pentose phosphate pathway, and the citric acid cycle. The cell decision-making to enter one of these pathways is coupled to redox transitions following nutrient availability. In the CCM, nutrients, such as glucose and glutamine, are used to generate precursors through redox reactions and to support cell growth. Moreover, experimental studies show that the mitochondrial activity is reduced during early progression in the cell cycle in G_1_ [32]. Also, the G_1_ phase of the cell cycle is characterized by an anabolic demand in protein synthesis, required for DNA replication in S phase. Synthesis of building blocks, such as amino acids and DNA or pyruvate from carbohydrate pathways is then a necessary step for biomass synthesis and energy supply through mitochondrial activity.

Moreover, one understands that cells decision-making to enter proliferation or stay in dormancy depends on physical, electrical and biochemical parameters. Nutrients and growth hormones availability in the extracellular medium modulates all these three parameters since they provoke osmotic pressure resulting in variation in bioelectrical parameters of the cell, such as the transmembrane potential, enzyme and cofactors charges and the intracellular (pHi). Perturbing one of these parameters has been reported to change the others. So that, cell metabolism seems to be the result of intertwined state parameter oscillations. In this literature investigation, we deciphered cell cycle progression from cell metabolism or more precisely central carbon metabolism (CCM) point of view. It appeared, first, the intriguing relationship between CCM and cell cycle progression, with the reactive and oxidative (redox) cofactors such as NAD^+^/NADH, NADP^+^/NADPH being key regulators. Secondly, as reported, mitochondria seem to be more than just a plant for ATP synthesis. They are at the core of eukaryotic cell metabolism and cell cycle progression. In there, the tricarboxylic acid (TCA) cycle, branched to glycolysis and to the pentose phosphate pathway, is central in mitochondrial metabolism and has been reported to match mitosis. The TCA is also an adaptive circuit at the crossroads between cytosolic-mitochondrial energy exchanges which are especially enhanced when resting cells are committed to divide. Finally, the progression of the cell cycle exhibits a shifted metabolism, materialized by a shunt from catabolism to anabolism. Transitions are performed by redox potential variation, involving NAD^+^/NADH, NADP^+^/NADPH redox couples, and ADP/ATP energetic ratios and the intracellular pH seems to the be master operator of cytosol/mitochondrial flux balances. Understanding the dynamics of these metabolic exchanges will pave the way to therapeutic solutions for metabolic cycle disorders such as cancer.

## Abbreviations

DNA: Deoxyribonucleic acid
RNA: Ribonucleic acid
NAD: Nicotinamide adenine dinucleotide
NADP: Nicotinamide adenine dinucleotide phosphate
ATP: Adenosine triphosphate
ADP: Adenosine diphosphate
CCM: Central carbon metabolism
TCA: Tricarboxylic acid
PPP: Pentose phosphate pathway
CO_2_: Carbon dioxide
ETC.: Electron transport chain
G3P: Glyceraldehyde 3-phosphate
ROS: Reactive oxygen species
F6P: Fructose 6-phosphate
G6PDH: Glucose-6-phosphate dehydrogenase
GSH: Glutathione
GSSH: Mitochondrial pyruvate carrier
FAD: Flavin adenine dinucleotide
NHE-1: Sodium-hydrogen exchanger 1
NaK-ATPase: Sodium-potassium ATPase
HATs: Histone acetyltransferases
HDACs: Histone deacetylase
